# Effectiveness of antipsychotic drug therapy for treating psychosis in people with epilepsy: A systematic review

**DOI:** 10.1111/epi.18123

**Published:** 2024-10-21

**Authors:** Aryan Arora, Priya Prakash, Laura Rizzo, Graham Blackman, Anthony S. David, Jonathan P. Rogers

**Affiliations:** ^1^ Medical School University College London London UK; ^2^ Division of Biosciences University College London London UK; ^3^ Department of Psychiatry University of Oxford Oxford UK; ^4^ Institute of Mental Health University College London London UK; ^5^ Division of Psychiatry University College London London UK

**Keywords:** antipsychotic, epilepsy, psychosis, seizure, systematic review

## Abstract

Individuals with epilepsy are at risk of developing preictal, ictal, postictal and interictal psychoses. Antipsychotic drugs (APDs) are the main class of drugs used to treat psychosis and schizophrenia. The efficacy and safety of APDs as a treatment for epileptic psychosis is not well understood. This systematic review aimed to assess the effectiveness and adverse effects of APDs for treating psychosis in people with epilepsy. We adhered to the PRISMA (Preferred Reporting Items for Systematic Reviews and Meta‐Analyses) guidelines. We searched MEDLINE, Embase, PsycInfo, and AMED (Allied and Complementary Medicine) from database inception to June 20, 2023. We contacted experts in the field and performed citation searches to identify additional records. Title, abstract, full‐text review, and data analysis were conducted in duplicate, with conflicts resolved by discussion among authors. Given the considerable heterogeneity of study designs, meta‐analysis was not deemed appropriate; instead, the results were tabulated in a narrative synthesis. The Joanna Briggs Institute Risk of Bias tool and GRADE (Grading of Recommendations Assessment, Development, and Evaluation) framework were used to assess study quality. We identified 13 studies with a total of 1180 participants. In the four case series included, the psychotic symptoms of 25 of 28 patients treated with APDs partially improved or fully resolved. Three of the four cohort studies reported an association between antipsychotic use and longer duration of psychotic episodes, two found similar results in both APD and non‐APD groups, and two did not report control psychosis outcomes. When reported, seizure frequency was observed to remain unchanged or decrease following APD treatment. The evidence on the effectiveness of antipsychotics in the treatment of psychosis in epilepsy is inconclusive and may reflect confounding by indication. However, most studies suggest that antipsychotics were not associated with a marked worsening in seizure frequency. It remains unclear whether antipsychotics should be used in epilepsy, and well‐controlled cohort studies and randomized controlled trials are necessary to draw definitive conclusions.

**Key points**

- Individuals with epilepsy are at risk of developing psychosis. Antipsychotics are the most commonly prescribed drugs to manage psychosis. Their effectiveness in people with epilepsy remains to be clarified.
- We conducted a systematic review of clinical studies to collect evidence on the effectiveness of APDs in individuals with epilepsy. We found four case series and nine cohort studies.
- Notably, there was considerable heterogeneity across the studies in terms of treatment protocols and psychosis outcome. APDs were largely effective in resolving symptoms of psychosis but were associated with longer hospital admissions. Seizure frequency, when reported, either remained unchanged or decreased following treatment with APDs.
- We suggest conducting randomized controlled studies or well‐controlled cohort studies to elucidate more definitively the effectiveness of APDs in people with epilepsy.

## 1 INTRODUCTION

Epilepsy denotes a group of seizure disorders affecting approximately 50 million people worldwide, conferring significant physiological, psychological, and social burdens.^1^ It is defined clinically by the International League Against Epilepsy as the occurrence of two or more unprovoked seizures occurring >24 h apart, or a single unprovoked seizure with a recurrence risk >60%.^2^ Individuals with epilepsy are at increased risk of developing psychosis (and vice versa).^3^, ^4^ In general terms, psychosis represents a complex and heterogeneous syndrome, characterized by the presence of hallucinations, delusions, and disorganized thoughts and behaviors. The specific causal relationship between psychosis and epilepsy remains disputed, but the comorbidity may represent either shared genetic vulnerabilities, neurotoxic processes, or a combination of both.^4^, ^5^ Psychoses in epilepsy are conventionally categorized based on their temporal relationship to seizures. Preictal, ictal, and postictal psychoses occur before, during, and after seizure activity, respectively, whereas interictal psychosis, which bears the strongest symptomatic resemblance to schizophrenia, has no clear relationship with seizure timing.^6^, ^7^ Other forms of epileptic psychosis can be related to treatment, with cases of de novo psychosis occasionally occurring after surgery,^8^, ^9^, ^10^ and “alternative psychosis” presenting in association with rapid electroencephalographic (EEG) normalization and seizure management using antiseizure medication (ASM).^11^, ^12^ Psychosis may also be iatrogenic, associated with ASM initiation, maintenance, or discontinuation.^13^ Lastly, although very rare, hypothalamic hamartomas present with refractory seizures, and occasionally with psychiatric symptoms including psychosis.^14^, ^15^, ^16^ The precise prevalence of epileptic psychosis is difficult to ascertain due to heterogeneity in both patient populations and diagnostic definitions between studies. A meta‐analysis conducted in 2014 estimates the pooled prevalence at 5.6% (95% confidence interval [CI] = 4.8–6.4), varying according to psychosis temporality, seizure type and location, and other co‐occurring conditions. Furthermore, the same review estimates the odds of having psychosis to be 7.8 (95% CI = 2.81–21.79) times higher in people with epilepsy compared to the general population.^17^

Antipsychotic drugs (APDs) are the main class of drugs used to treat psychosis and schizophrenia. They fall into two major classes: typical (first‐generation/conventional) antipsychotics and atypical (second‐ and third‐generation) antipsychotics. Both classes are broadly considered to exert their effects principally via the blockade of dopaminergic D2 receptors.^18^ Second‐generation antipsychotics additionally block 5HT2A serotonergic receptors.^19^ Antipsychotics may induce a range of side effects. However, these differ based on the type of APD used, with some patients experiencing extrapyramidal symptoms as well as endocrinological, cardiovascular, and metabolic derangements.^20^

Although antipsychotics are sometimes used to manage irritability and aggression in epilepsy,^21^, ^22^ this review focuses on their use in the treatment of people with psychosis and epilepsy—an unresolved controversy in the field.^23^ Some antipsychotics are considered proconvulsive and have been thought to lower the seizure threshold in people with epilepsy. A study by Lertxundi et al.^24^ on antipsychotic‐related seizures found that second‐generation antipsychotics carry a higher seizure risk, with clozapine carrying the highest risk despite being the most effective antipsychotic.^25^ However, other studies of nonepileptic patients have shown that there is little to no evidence of APDs inducing seizures.^26^, ^27^ Another potential risk of antipsychotic use in people with epilepsy is drug–drug interaction with ASMs. The coprescription of antipsychotics that are metabolized by the enzyme CYP3A4 with antiepileptic drugs that are CYP3A4 inducers can result in significantly reduced plasma concentrations of the antipsychotic.^23^ For instance, quetiapine doses up to 700 mg have been reported as undetectable in blood–serum in combined carbamazepine treatment.^28^ Drug–drug interactions can also lead to elevated concentrations of toxic metabolites.^29^ Aside from antipsychotics, other medications such as benzodiazepines are sometimes employed in the management of epileptic psychosis, with varying outcomes.^23^

Several nonsystematic literature reviews and consensus statements from internal experts have attempted to synthesize the available information on antipsychotic prescription in this population.^10^, ^23^, ^30^, ^31^, ^32^, ^33^, ^34^, ^35^ To our knowledge, the only systematic review of APDs in epilepsy is a Cochrane review conducted in 2014.^36^ This review limited its eligibility criteria to randomized controlled trials (RCTs) and found a single study, published as a conference abstract, that met eligibility. This study compared the effects of olanzapine and haloperidol in people with schizophrenia‐like psychosis in epilepsy and included only 16 participants, 13 of whom completed the study. Compared to the haloperidol group, treatment with olanzapine was associated with a greater reduction in psychotic symptoms. Additionally, seizure frequency was reported as unchanged in the olanzapine group, but increased from baseline among patients treated with haloperidol. Given the paucity of RCT evidence, it is reasonable to examine observational studies to support clinical decision‐making. Accordingly, our systematic review aimed to more broadly survey the existing evidence on the effectiveness and safety of antipsychotics in the treatment of psychosis in individuals with epilepsy.

## 2 MATERIALS AND METHODS

### 2.1 PICOS statement

The review question was formulated using the PICOS framework, which defines the Population, Intervention, Comparison, Outcome, and Study Design of eligible studies (see Table 1 for the full PICOS statement).

**TABLE 1:** PICOS statement.

Population	Individuals with both epilepsy and psychosis
	Any age, gender, and ethnicity
Intervention	Antipsychotic medications (WHO ACC N05A listed)^39^
Comparison	Placebo, treatment as usual or waitlist
Outcome	Primary outcome: psychosis severity or duration of hospitalization
	Secondary outcomes: seizure frequency, quality of life, adverse events
Study design	Randomized controlled trials, cohort studies, case–control studies, and case series in human participants with five or more participants

Abbreviations: PICOS, population, intervention, comparison, outcome, study design; WHO, World Health Organization. ACC, Anatomic Theraputic Chemical

### 2.2 Protocol and registration

The review protocol was registered prospectively with PROSPERO (CRD42013006322). Our review was compliant with the Preferred Reporting Items for Systematic Reviews and Meta‐Analysis (PRISMA) statement^38^ (see Table S1).

### 2.3 Search strategy and study selection

We searched MEDLINE, Embase, PsycInfo and AMED (Allied and Complementary Medicine; via Ovid) from database inception to June 20, 2023. Gray literature sources (via OpenGrey) were also searched. Retrieved studies were uploaded and managed in Covidence.^39^ The search strategy consisted of a combination of search terms denoting “epilepsy,” “psychosis,” and “antipsychotic” medications. The full search strategy can be found in Table S2. Three authors (A.A., P.P., and L.R.) independently screened titles and abstracts with blinding between authors until all studies had been evaluated by two authors. Conflicts were resolved via discussion, with the third reviewer making the final decision. Full‐text screening followed a similar process, but disputes were discussed with a fourth author (J.P.R.). We identified additional studies by contacting experts in the field and conducting forward/backward citation searching from the included studies.

### 2.4 Eligibility criteria

Studies published in peer‐reviewed journals were considered eligible if individuals diagnosed with epilepsy had evidence of psychosis and at least some were treated with APDs. APDs were defined as any medication included in the World Health Organization Anatomical Therapeutic Chemical/Defined Daily Dose database, indexed under the code N05A.^37^ Studies were not excluded based on comorbid diagnoses, as this would bias the sample away from real‐world clinical experience. Eligible study types were randomized controlled trials, cohort studies, case–control studies, and case series.

We excluded secondary research, studies with fewer than five participants, studies with nonhuman participants, and those with full text not in English, Italian, or Hindi. Studies that consisted only of participants with functional seizures (also known as psychogenic nonepileptic seizures) were also excluded.

### 2.5 Data extraction

Three authors (A.A., P.P., and L.R.) independently extracted data from the included studies, with each study evaluated by two authors. The extraction forms were compared and cross‐checked by the third author, resolving disputes and ensuring accuracy.

Based on an a priori extraction template, the following data were extracted from each study: general publication data (lead author, publication date, country of study, study design, and conflicts of interest), study characteristics (study duration, temporality, total sample size, number of individuals exposed and unexposed to antipsychotics, follow‐up duration, antipsychotic type, antipsychotic dosage, eligibility criteria, psychosis rating scale, and definition of intervention groups), baseline population characteristics (age, ethnicity, sex, epilepsy diagnosis, psychosis type, psychosis severity, and seizure frequency), and outcomes (seizure frequency and psychosis severity).

Study methodologies were defined based on the relevant case definition criteria referenced in Appendix 1 of the JBI Risk of Bias Checklist Tools.^40^, ^41^

### 2.6 Outcome measures

The primary outcome was the effectiveness of APDs in treating psychosis in individuals with epilepsy. The secondary outcome was frequency of seizures and any side effects of APD therapy. Both outcomes were either defined by the study or based on changes detected using a validated screening tool.

### 2.7 Risk of bias assessment

The appropriate Joanna Briggs Institute (JBI) Risk of Bias tool was used to assess included studies for methodological quality and reliability.^40^, ^41^ Each study was assessed by two authors and scored on a scale of “high,” “moderate,” or “low” for risk of bias, with the third checking for consensus. Unresolved discrepancies were discussed with J.P.R.

### 2.8 Quality of evidence

We assessed the certainty of evidence for our primary and secondary outcomes using the Grading of Recommendations Assessment, Development, and Evaluation (GRADE) framework. Ratings were based on the following criteria: risk of bias, inconsistency, indirectness of evidence, imprecision, publication bias, large effect size, possible confounding, and dose–response relationships as stipulated by the GRADE handbook. Specific decisions regarding judgments are recorded in Tables 2 and 3.

**TABLE 2:** GRADE ratings for case series.

Certainty assessment							Certainty
Outcome	Study design	Risk of bias	Inconsistency	Indirectness	Imprecision	Other considerations	
Psychosis severity	Case series	Very serious^a^	Not serious	Not serious	Serious^b^	Publication bias strongly suspected^d^	⊕ ⊖ ⊖ ⊖ Very low
Seizure frequency	Case series	Very serious^a^	Not serious	Not serious	Very serious^b^, ^c^	Publication bias strongly suspected^d^	⊕ ⊖ ⊖ ⊖ Very low

Abbreviation: GRADE, Grading of Recommendations Assessment, Development, and Evaluation.

^a^ Lack of standardized rating instruments used; reports may be based on circumstantial evidence.

^b^ Small sample sizes.

^c^ Strong suspicion of publication bias due to the possibility that only positive or significant findings are published.

^d^ Data not available for all participants who received intervention, contributing to very serious imprecision.

**TABLE 3:** GRADE ratings for cohort studies.

Certainty assessment							Certainty
Outcome	Study design	Risk of bias	Inconsistency	Indirectness	Imprecision	Other considerations	
Psychosis severity	Cohort	Serious^a^	Serious^b^	Serious^c^	Not serious	None	⨁◯◯◯ Very low
Seizure frequency	Cohort	Serious^a^, ^d^	Not serious	Not serious	Not serious	None	⨁◯◯◯ Very low

Abbreviation: GRADE, Grading of Recommendations Assessment, Development, and Evaluation.

^a^ Lack of control arm in studies, lack of demographic information.

^b^ Inconsistent outcomes for duration of illness and length of hospitalization.

^c^ Lack of standardized validated rating scale use, length of hospital stay and duration of illness used as proxy for severity.

^d^ Most included studies did not report seizure outcomes.

Observational studies were initially rated as low certainty. These studies were then downgraded if they met any of the first five criteria listed above (risk of bias, inconsistency, indirectness of evidence, imprecision, or publication bias), and upgraded if they met any of the last three criteria (large effect size, possible confounding, or dose–response relationships). Ratings were determined through discussions involving A.A., P.P., and L.R., and were verified by J.P.R. The assessments were performed, and summary tables generated using GRADEpro GDT software.^42^

## 3 RESULTS

### 3.1 Study description

**FIGURE 1:**
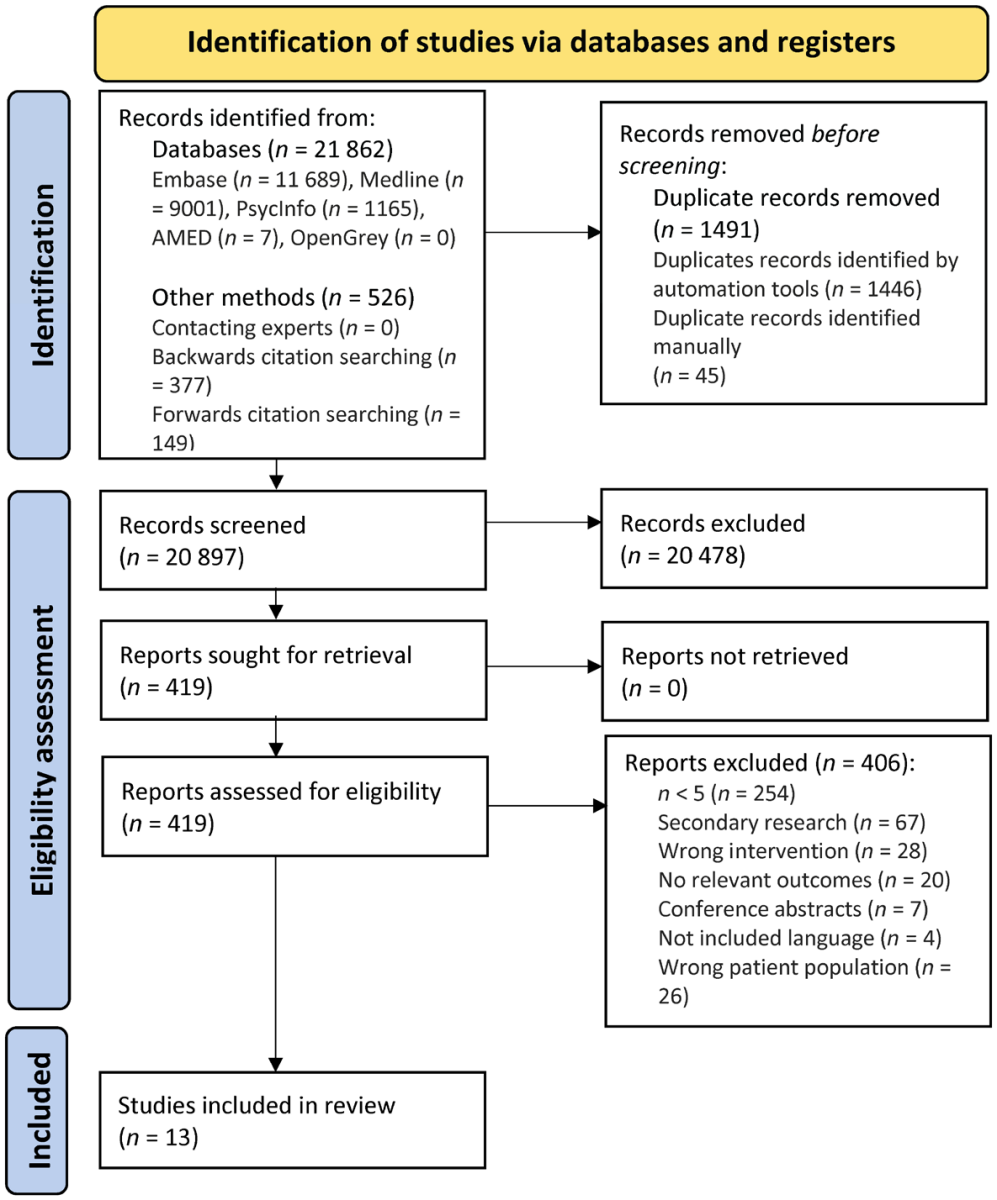
PRISMA (Preferred Reporting Items for Systematic Reviews and Meta‐Analyses) flow diagram. A visual summary is provided of the screening process for studies identified using the search strategy, showing the total number of reports initially identified, as well as those that were included or excluded, with reasons for exclusion provided.

Our database search retrieved 21 862 results, of which 406 studies were assessed for eligibility, leaving 13 studies included in our systematic review. The PRISMA flow diagram is shown in Figure 1. Of the included studies, we identified four case series and nine cohort studies. A total of 1180 participants were identified. The mean age of participants across studies was 33 years (SD = 6), with 600 male (51%) and 580 female participants (49.15%). The route of APD administration was not stated in any study. Information about study characteristics, including location and eligibility criteria, is provided in Tables S3a and S3b.

## 4 CASE SERIES

There were four case series reporting results on 28 individuals. These are summarized in Table 4. Using the JBI critical appraisal tool for case series, the risk of bias was considered to be high in one of these studies,^45^ moderate in two,^43^, ^44^ and low in one^46^ (see Table S4a for full quality assessment). The diagnostic groups were as follows: interictal psychosis (*n* = 13), postictal psychosis (*n* = 2), schizophrenia (*n* = 7), Korsakoff syndrome (*n* = 1), atypical psychosis (*n* = 4), and organic psychosis (*n* = 1).

**TABLE 4:** Characteristics and outcomes of case series.

Study	*n*	Psychosis type or phenomenology (*n*)	Epilepsy or seizure type (*n*)	Age, years, mean, SD; sex, males, females	Antipsychotic type	Psychosis outcome (*n*)	Seizure frequency outcome (*n*)	Other adverse effects and complications
Blumer et al., 2000 ^43^	10	Interictal psychosis (10)	Complex partial seizures (3) Complex partial and generalized seizures (7) Generalized seizures (9)	28.6, 11.4; 7 M, 3 F	Fluphenazine, loxapine, trifluoperazine, risperidone, haloperidol, chlorpromazine	Psychosis remitted (3) Treatment partially effective, but clinical improvement incomplete or transient (4) Treatment ineffective (3)	NR	1 case of drug‐induced parkinsonism
Langosch & Trimble, 2002^44^	6	Interictal psychosis (3), Schizophrenia (2) Korsakoff psychosis (1)	Partial seizures (5) Grand mal (1)	36.3, 11.1; 2 M, 4 F	Clozapine	Psychosis improved in all patients	No overall change (3) Seizure during clozapine initiation, then seizure‐free for >3 years (1) Decrease in seizure frequency (2)	1 case of neutropenia after 18 months on clozapine. Second trial of clozapine lasted 3 years before discontinuation for further neutropenia.
Leinonen et al., 1994^45^	5	Postictal psychosis (2) Schizophrenia (3)	TLE, partial seizures	38.4, 4.5; 1 M, 4 F	Perphenazine, haloperidol, thioridazine, chlorpromazine	Psychosis severity decreased in all patients	1 patient experienced a cluster of 6 seizures after surgery and thioridazine resumption Another patient experienced a 30‐min‐long generalized seizure weeks after starting 3 mg/day haloperidol and 150 mg/day chlorpromazine	Perphenazine dosage had to be reduced due to fatigue
Pakalnis et al., 1987 ^46^	7	Atypical psychosis (4) Organic psychosis with thought disorder (1) Paranoid schizophrenia (2)	Complex partial epilepsy (3) Complex partial epilepsy with secondary generalization (3) Absence seizures with primary generalization (1)	30.1, 8.76; 2 M, 5 F	Haloperidol	Psychosis resolved in all cases	No increase in seizure frequency observed, ceased in most patients with concurrent treatment with ASMs	NR

Abbreviations: ASM, antiseizure medication; F, female; M, male; NR, not reported; TLE, temporal lobe epilepsy.

Psychosis was reported as resolved or remitted in 10 of the 28 patients treated with antipsychotics and improved in 15 (with four of these qualified as incomplete/transient improvements), and treatment was ineffective in three. Overall, the psychosis severity outcome received a very low GRADE certainty of evidence score across the case series.

In three of four case series, seizure frequency did not change for the majority of patients treated with antipsychotics. However, one patient experienced a seizure during clozapine initiation (followed by >3 seizure‐free years),^44^ and Leinonen et al.^45^ reported a patient who experienced a cluster of six seizures after surgery and thioridazine resumption, as well as a patient who experienced a 30‐min seizure, weeks after haloperidol and chlorpromazine administration. The fourth case series, Blumer et al.,^43^ did not report the impact of antipsychotic medication on seizures. Overall, the seizure frequency outcome received a very low GRADE certainty of evidence score across the case series.

Three patients were noted to have experienced other adverse effects, including neutropenia following clozapine initiation and continuation,^44^ fatigue,^45^ and drug‐induced parkinsonism.^43^

## 5 COHORT STUDIES

There were nine cohort studies, reporting results on 1152 participants. These are summarized in Table 5. Using the JBI critical appraisal tool for cohort studies, five of these^48^, ^49^, ^51^, ^54^, ^55^ were considered to have a low risk of bias and four to have a moderate risk of bias^47^, ^50^, ^52^, ^53^ (see Table S4b for full quality assessment). The diagnostic groups were as follows: interictal psychosis (*n* = 527), postictal psychosis (*n* = 211), antiepileptic‐induced psychotic disorder (*n* = 14), schizophrenia (*n* = 134), paranoid psychosis (*n* = 15), “obsessive–compulsive psychosis” (*n* = 1), and psychosis unspecified (*n* = 250). Comparison groups varied; seven studies included patients with psychosis and epilepsy who were not given antipsychotics (although two of these did not present results for the comparison group).^50^, ^53^ One study included patients with epilepsy but without psychosis who were not given antipsychotics,^54^ and one study included patients without epilepsy but with schizophrenia who were given antipsychotics.^55^ The number of psychotic episodes was reported in only two studies,^47^, ^51^ where antipsychotics were not used in 146 of the reported episodes and were administered in 718 episodes (of which antipsychotics were given prophylactically in 59). In the seven studies that reported follow‐up, the mean follow‐up time was 101.5 months (SD = 84.2).

**TABLE 5:** Characteristics and outcomes of cohort studies.

Study	*n*	Psychosis type or phenomenology (*n*)	Epilepsy type (*n*)	Age, years, mean, SD; sex, males, females	Antipsychotic type, mean dose	Intervention groups (*n*)	Psychosis outcome (*n*)	Seizure frequency outcome (*n*)	Other adverse effects
Adachi et al., 2012^47^	155 (320 episodes)	IIP	Partial epilepsy (130): TLE (69), FLE (31), PLE (7), OLE (6), multilobe/ undetermined lobe epilepsy (14), benign epilepsy of childhood with centrotemporal foci (3); Generalized epilepsy (25)	Age of psychosis onset: 30.9, 10.5; 89 M, 66 F	NR	Non‐APD	Mean duration of IIP episodes: 28.9 weeks	NR	NR
						APD prophylactic [taken before the onset of recorded PIP episode, treatment continued throughout episode]	Mean duration of IIP episodes: 59.1 weeks	NR	NR
						APD add‐on [treated with APDs after psychosis onset, treatment continued throughout episode]	Mean duration of IIP episodes: 117.6 weeks	NR	NR
Adachi et al., 2007^48^	58	PIP; 13 patients had an additional history of IIP during the course of epilepsy	Localization‐related epilepsy: TLE (46), FLE (7), OLE (2), multilobular/epilepsy of undetermined location (3)	Age of psychosis onset: 34.1, 13.5; 32 M, 26 F	Antipsychotic type NR, mean dosage as haloperidol equivalents .5 mg/day or more	APD prophylactic [taken before the onset of recorded PIP episode, treatment continued throughout episodes]	Mean duration of PIP episodes: lower than mean	NR	NR
						APD add‐on [treated with APDs after psychosis onset, treatment continued throughout episode]	Mean duration of PIP episodes: higher than mean	NR	NR
						Non‐APD	Mean duration of PIP episodes: lower than mean	NR	NR
Chen et al., 2016^49^	14	AIPD	Generalized seizures (3), focal seizures (11) Lateralization: left (6), right (5), bilateral (3)	40.93, 16.41; 5 M, 9 F	Droperidol, haloperidol, olanzapine, risperidone	Olanzapine (4)	2 relapses, 2 single episodes	NR	NR
						Risperidone (3)	1 relapse, 2 single episodes	NR	NR
						Droperidol, haloperidol (1)	1 relapse	NR	NR
						Non‐APD (6)	2 relapses, 4 single episodes	NR	NR
Hamed & Attiah, 2019^50^	146	IIP; 112 patients had history of PIP	TLE (73), FLE (62), GTC (11)	39.25; 46 M, 100 F	Aripiprazole: (10–30 mg/day), haloperidol (10–30 mg/day), olanzapine (5–20 mg/day), quetiapine (25–100 mg/day)	APD	Rapid and complete resolution of psychotic symptoms followed by gradual withdrawal of APDs	NR	NR
						Non‐APD	NR	NR	NR
Hara et al., 2013^51^	200 (338 episodes)	IIP; 9 patients had a history of PIP	Partial epilepsy (172): TLE (91), FLE (45), PLE (8), OLE (6), multilobular/undetermined lobe epilepsy (19), benign epilepsy of childhood (3); generalized epilepsy (28): IGE (19), SGE (6), special epilepsy syndromes (3)	31.0, 10.4; 107 M, 93 F	FAPDs: butyrophenone, phenothiazines, benzamides; SAPDs: serotonin–dopamine antagonists, dibenzothiazepines, multiacting receptor‐targeted antipsychotics, dopamine system stabilizers	Non‐APD	Mean duration of IIP episodes: ~28 weeks	NR	NR
						APD	Mean duration of IIP episodes: ~98 weeks	NR	NR
						FAPD	Mean duration of IIP episodes: ~80 weeks	NR	NR
						SAPD	Mean duration of IIP episodes: ~78 weeks	NR	NR
						CAPD	Mean duration of IIP episodes: ~225 weeks	NR	NR
Kara & Ünlüer, 2017^52^	32	PIP	Generalized seizures (29), focal seizures (3)	39, 15.3; 19 M, 13 F	Haloperidol	Haloperidol	Percentage of patients discharged: 71.4%	Seizure recurrence (1)	NR
						Benzodiazepine	Percentage of patients discharged: 72.2%	Seizure recurrence (4)	NR
						No medication	Percentage of patients discharged: 71.4%	Seizure recurrence (2)	NR
Kubagawa et al., 1997^53^	16	Paranoid (15), obsessive–compulsive (1)	IGE (7), CLE (9)	21.94, 8.86; 3 M, 13 F	Haloperidol	Haloperidol	Effect of treatment on psychosis classified as positive (11)	NR	NR
						Non‐APD	NR	NR	NR
Okazaki et al., 2014^54^	459	Schizophrenia and related disorders (75), nonpsychotic disorders, i.e., affective, neurotic, personality, and developmental disorders (75)	IGE (12); partial epilepsy (138): TLE (64), FLE (52), PLE (2), OLE (3), multilobular with unknown foci (16)	32.1, 10.4; 252 M, 207 F	Chlorpromazine equivalents noted, 662.7 CPeq mg/day	APD	5 patients with psychotic disorders rediagnosed as nonpsychotic	Increase in frequency (5); unchanged frequency (87); decrease in frequency (39)	NR
						Non‐APD	NR	Increase in frequency (13); unchanged frequency (271); decrease in frequency (25)	NR
Tadokoro et al., 2007^55^	72	IIP (13), schizophrenia (59)	TLE (6), FLE (1), IGE (1), nonsymptomatic localized epilepsy (5)	27.9, 10.9; 35 M, 37 F	Risperidone equivalents noted, 2.25 mg/day in IIP group, 3.08 mg/day in schizophrenia group	APDs in IIP patients	1 year after treatment, mean PANSS scores decreased from 59.5 [SD = 16.9] to 38.7 [SD = 4.6], 77.8% responder rate	Overall decrease in seizure frequency: weekly seizures (1), monthly (2), yearly (3), in remission (7)	NR
						APDs in schizophrenia patients	1 year after treatment mean PANSS scores decreased from 80.9 [SD = 16.3] to 52.9 [SD = 12.9], 76.2% responder rate	NR	NR

Abbreviations: AIPD, antiepileptic drug‐induced psychotic disorder; APD, antipsychotic drug; CAPD, combination of first‐ and second‐generation antipsychotic drugs; CLE, cryptogenic localization‐related epilepsy; CPeq, chlorpromazine equivalent; F, female; FAPD, first‐generation antipsychotic drug; FLE, frontal lobe epilepsy; GTC, generalized tonic–clonic; IGE, idiopathic generalized epilepsy; SGE, symptomatic generalized epilepsy; IIP, interictal psychosis; M, male; NR, not reported; OLE, occipital lobe epilepsy; PANSS, Positive and Negative Syndrome Scale; PIP, postictal psychosis; PLE, parietal lobe epilepsy; SAPD, second‐generation antipsychotic drug; TLE, temporal lobe epilepsy.

Due to methodological heterogeneity, meta‐analysis was not deemed to be appropriate. Of the nine studies, five clearly reported psychosis outcomes for non‐APD control groups,^47^, ^48^, ^49^, ^51^, ^52^ three did not,^50^, ^53^, ^54^ and one did not have a non‐APD control group.^55^ When compared against non‐APD groups, three of these studies reported that antipsychotic use was associated with a longer duration of psychotic episodes.^47^, ^48^, ^51^ Further comparisons found the duration of interictal psychosis (IIP) episodes to be shorter when APD use began before (prophylactic) rather than after (add‐on) the onset of the episode.^47^, ^48^ Hara et al.^51^ observed similar psychosis outcomes for patients treated with first‐generation and those treated with second‐generation APDs, whereas patients receiving a combination of the two (CAPD) experienced far longer IIP episodes; the paper noted that CAPD groups were given much higher doses of antipsychotics, which may have contributed to this observation.^51^ Kara and Ünlüer^52^ measured the psychosis outcome as the number of patients who were discharged, rather than those hospitalized after treatment, recording a marginally higher discharge rate (72.2%) in patients treated with benzodiazepines as compared to those treated with APDs and those who did not receive treatment. APD and no‐treatment groups had identical discharge rates of 71%. Chen et al.^49^ reported similar psychosis outcomes in APD and non‐APD groups. The psychosis severity outcome received a very low GRADE certainty of evidence score across the cohort studies.

Tadokoro et al.^55^ used different intervention groups, noting a better response to APDs in patients with IIP compared with schizophrenia patients without epilepsy. However, this purported intergroup difference was nullified once dropouts were accounted for.

Seizure frequency following APD treatment was reported in three cohort studies.^52^, ^54^, ^55^ Only two out of these of these three studies reported the seizure frequency outcomes for non‐APD groups.^52^, ^54^ One of these, Okazaki et al., observed a higher number of patients with decreased seizure frequency and a lower number of patients with increased seizure frequency in the APD group than in the non‐APD group.^54^ The other, Kara and Ünlüer, reported similar seizure recurrence rates for haloperidol and non‐APD groups.^52^ The seizure frequency outcome received a very low GRADE certainty of evidence score across the cohort studies.

No other adverse effects were reported or explicitly investigated in the included studies.

## 6 DISCUSSION

This systematic review analyzed 13 studies investigating the use of APDs in 1180 individuals with alternative, interictal, or postictal epilepsy‐related psychoses. Overall, the case series reported that the vast majority of patients exhibited improvement in psychosis symptoms following APD administration. Of the five cohort studies that reported control group outcomes, APD groups performed worse than non‐APD groups in three studies^47^, ^48^, ^51^ and had similar effects in two.^49^, ^52^ No evidence was found to suggest an increase in seizure frequency following antipsychotic administration.

In the four case series identified,^43^, ^44^, ^45^, ^46^ the majority of the 28 patients observed experienced an improvement in psychosis severity or complete remission of symptoms following antipsychotic use, with the exception of three patients for whom treatment was reported to be ineffective.^43^

Of the nine cohort studies included, five reported non‐APD group psychosis outcomes. Two of five studies observed similar psychosis outcomes in APD and non‐APD groups,^49^, ^52^ although one of these noted that benzodiazepines performed marginally better (72.2% success rate compared to 71.4% in both the no‐treatment and the haloperidol group).^52^ The remaining three studies^47^, ^48^, ^51^ observed APD groups as performing worse than non‐APD groups, recording a longer average duration of postictal psychotic episodes in those treated with antipsychotics compared to control groups. Adachi et al.^47^, ^48^ further separated the APD group into prophylactic, where APDs were administered before the onset and continued throughout the observed postictal psychosis episode, and add‐on, where APDs were administered after the onset and continued throughout the episode. They found that the average duration of psychotic episodes was shorter in those treated prophylactically, suggesting that the timing of treatment administration may affect the response to antipsychotic medication.

In those cohort studies that considered seizure frequency as an outcome,^52^, ^54^, ^55^ there was no evidence to suggest an increase in frequency following antipsychotic treatment. The majority of individuals included in the case series also did not experience increased seizure frequency, with only three of 28 patients reported as having further seizure activity following treatment administration.

Other adverse effects such as neutropenia, fatigue, and parkinsonian states were observed following treatment with antipsychotic medication in a small number of cases.^43^, ^44^, ^45^

### 6.1 Strengths and limitations

Our systematic review was both rigorous and comprehensive, following the PRISMA checklist and reducing the risk of bias by ensuring three decisions, two blinded and one consensus, were made on each study. Preregistration of the review further minimized bias in reporting and interpretation of the available information. We designed our inclusion criteria to be broad and liberal, considering multiple study types to provide insight into the existing evidence landscape and allow us to capture an ecologically valid patient population.

The limitations of this review can largely be attributed to the lack of high‐quality evidence regarding antipsychotic use in cases of epileptic psychosis. Heterogeneity existed not only on an interstudy level, but also on an intrastudy level, with case series using varying follow‐up periods and outcome measures, and cohort studies investigating populations with a range of epilepsy types and baseline psychosis severities. Further variation was seen in the type and dose of antipsychotic treatment, with a lack of consistent reporting of prescription schedule and drug titration. Variation was present even between the comparison groups used, with most studies comparing different treatment strategies, and one comparing the same medication used in different populations.^55^ This heterogeneity, as well as the lack of both robust, objective outcome measures and statistical reporting amongst studies, meant they were deemed unfit for meta‐analysis, and instead required qualitative evidence synthesis. Some studies failed to provide control groups or, as in the case of Hamed and Attiah,^50^ did not report control outcomes. This limited our ability to make conclusions regarding the effectiveness of antipsychotics as compared to alternative drug therapies and supportive clinical management. An important methodological limitation was restricting eligibility by language, as this may have introduced geographical biases in the inclusion of studies. Based on United Nations classifications, none of the included studies come from “medium,” “low,” or “very low” income countries, which may also reflect a publication bias in the broader literature. Finally, our risk of bias assessment would perhaps benefit from domain‐based measures, such as ROBINS‐I (Risk of Bias in Non‐Randomized Studies of Interventions), in line with contemporary best practice.

In general, a more convincing relationship between antipsychotic treatment and psychosis remission was seen in data from case series than that from cohort studies. Although reporting and selection bias may have contributed to this observation, another plausible explanation for this discrepancy is confounding by indication. This results from the possibility that antipsychotics may have been preferentially prescribed in the more severe or persistent cases of psychosis, resulting in APD groups having fewer positive outcomes than control groups. This particular source of bias was identified in a minority of the cohort studies used, but was not accounted for.^51^, ^55^ Most of the studies were based on relatively small sample sizes, which may have failed to adequately represent the broad phenomenological landscape of psychosis in epilepsy. Such small pilot studies are also unlikely to detect the rarer adverse effects of antipsychotics, such as cardiac arrhythmias or respiratory complications. Another limitation worth considering is regression toward the mean; in patients with severe psychosis or particularly high seizure frequency, reported improvement in symptoms may have partially been a result of this phenomenon, which could overstate the impact of APDs in the case series. This should be considered in those included studies that reported psychosis and seizure frequency outcomes as changes from baseline, that is, improvements in psychotic symptoms or increased/decreased seizure frequency.

### 6.2 APDs and seizure outcomes

Considering the literature more broadly, the effects of antipsychotics on seizure frequency in people with epilepsy remains equivocal. Certain atypical APDs are associated with an increased seizure risk.^24^ Three structurally related atypical APDs (clozapine, olanzapine, and quetiapine) have been linked to an increased prevalence of seizures compared to placebo in a large meta‐analysis,^30^, ^56^ although the evidence is clearest for clozapine. Analysis of UK‐based primary care cohorts suggests that the rates of seizures were not significantly different for olanzapine and quetiapine compared to other atypical antipsychotics.^57^ However, in people with dementia, seizure rates were significantly elevated.^58^ This implies that extrapolating evidence from individuals without existing neurological disease may not always be appropriate. We should note that the statistical power of the studies included in our review is considerably less than that of larger studies of seizure risk in the general population, meaning that there may be smaller effects we would be unable to observe.

Certain factors, such as APD initiation even at low doses, maintenance at high doses, and duration of exposure, have been associated with increased seizure incidence both in people with and those without epilepsy, affecting individuals with epilepsy to a greater extent.^59^, ^60^, ^61^ The relationship of seizure frequency with dose or speed of titration remains unclear, although large dose escalations and altered drug–drug interactions are thought to increase risk of seizures.^58^, ^62^

Mechanistically, clozapine has been shown to attenuate chloride currents in isolated neural tissue^63^ and generate aberrant electrophysiology in induced pluripotent stem neural cell lines.^64^ In humans, clozapine and olanzapine have been associated with EEG abnormalities.^56^, ^62^ However, the significance of these findings in the pathogenesis of APD‐related seizures in humans remains unclear. Clozapine has also been shown to prolong seizures in electroconvulsive therapy, providing indirect evidence of its role in seizure pathology.^65^

It also remains uncertain whether seizures following APD administration can be attributed solely to the APDs or to an individual's underlying epilepsy vulnerability, because most studies do not control for this.^66^ The adjunctive use of ASMs appears essential for effective seizure control, as they have demonstrated strong potential for seizure management^58^ even in the presence of APDs, as indicated by Langosch and Trimble.^44^

Weighing the risk between psychiatric symptoms and seizure outcomes is challenging due to the complex and reciprocal relationship between them. Improving psychosis outcomes, for instance, may increase compliance with ASMs, leading to better overall seizure control.^66^ Alternatively, improved seizure control may extinguish psychiatric sequelae.

### 6.3 Future research

This review has several important implications. Regarding research, there is an urgent need for high‐quality randomized controlled trial evidence of APDs for psychosis in epilepsy, especially because included studies suggest there may be a possibility of APDs worsening outcomes in people with epilepsy, notwithstanding the issue of confounding by indication. Given that several acceptably sized nonrandomized studies have already been conducted, it seems feasible that an adequately powered trial may be performed. Multisite international collaborations involving low and middle income countries, where incidences of psychosis^67^ and epilepsy^68^ remain prevalent, may be beneficial in generating adequately powered and representative studies. In lieu of an RCT, a large cohort study would also provide useful comparative data.

A major limitation of the included studies is the lack of validated rating scales when measuring changes in epilepsy and psychosis outcomes. Future research should employ tools such as the Positive and Negative Syndrome Scale, which is most commonly used to quantify and track changes in psychotic symptoms over time, despite its limitations in capturing cognitive, motivational, and functional deficits,^69^ which may be highly represented in epileptic psychosis.^7^ As highlighted in a recent review,^70^ several rating scales for epilepsy outcomes have also been developed, such as the National Hospital Seizure Severity Scale^71^ and the Personal Impact of Epilepsy Scale.^72^ The latter of these considers multiple dimensions of epilepsy severity, incorporating quality of life, adverse events, and seizure impact measures.^70^ Use of such scales will facilitate a more robust and objective assessment of APD efficacy and safety across studies, allowing researchers to adjust analyses for the severity of psychotic symptoms.

### 6.4 Clinical implications

Prescribers can be reassured that most antipsychotics do not appear to increase seizure risk.^44^, ^45^, ^46^, ^52^, ^54^, ^55^ This statement is qualified by the possibility that poor case definition, inadequate monitoring, and incomplete reporting meant that adverse events may have not been captured precisely or reliably.^73^ We found no evidence favoring any particular antipsychotic, and it seemed that those selected in studies were not done so systematically, but instead based on pragmatic considerations and clinician preferences. Interestingly, results from Adachi et al.^47^, ^48^ suggest a greater improvement in psychosis outcomes when antipsychotics are administered before, rather than after, the onset of psychotic episodes, stressing the importance of early identification and management of psychosis in this population.^74^ Given the lack of data on APD efficacy in people with epilepsy, guidelines emphasize the importance of a multidisciplinary approach involving epileptologists, psychiatrists, psychologists, and other health care professionals. In some cases, appropriate seizure control may be sufficient to treat ictal and postictal psychosis; however, further psychopharmacological treatment is sometimes necessary.^66^ In this instance, high‐potency typical APDs, along with risperidone, amisulpride, and aripiprazole, have been suggested to have the lowest risk of seizure exacerbation, whereas low‐potency typical APDs such as chlorpromazine, loxapine, and zotepine are considered among the highest risk. Due to complex pharmacokinetics and the inability to withdraw quickly, depot antipsychotic formulations are also considered inappropriate in epileptic psychoses.^75^

Psychosis in epilepsy is a severe comorbidity. Therefore, until further data or alternative treatments emerge, antipsychotics still stand as a reasonable approach for managing psychosis in epilepsy, given careful monitoring and sound clinical judgment.

## 7 CONCLUSIONS

In summary, there is scant high‐quality and reliable evidence on the use of antipsychotics in epilepsy. Thus, no definitive conclusions can be drawn regarding the effectiveness or safety of these drugs, but the available evidence suggests that they are unlikely to substantially increase seizure frequency in the management of psychotic disorders in epilepsy. The field would benefit from the execution of a well‐designed RCT or a cohort study carefully adjusting for baseline confounding variables.

## AUTHOR CONTRIBUTIONS

Aryan Arora, Priya Prakash, and Laura Rizzo contributed equally to refining the research question, executing methodological procedures, and writing of the manuscript. Jonathan P. Rogers conceptualized the research question and methodology and assumed a supervisory role in all stages of this study. Jonathan P. Rogers, Graham Blackman, and Anthony S. David provided feedback on the manuscript and supported with the interpretation of results.

## FUNDING INFORMATION

J.P.R. was funded by the Wellcome Trust and NIHR at the time of this study. P.P. received a stipend from the Laidlaw Foundation to undertake this work.

## CONFLICT OF INTEREST STATEMENT

J.P.R. reports research funding from the Wellcome Trust and NIHR; royalties from Taylor & Francis; payment for reviewing from Johns Hopkins University Press; and speaker fees from the Alberta Psychiatric Association, Infomed Research & Training, North East London NHS Foundation Trust, and Vanderbilt Medical Center. He is a council member for the British Association for Psychopharmacology, a member of the Medical Advisory Board of the Catatonia Foundation and an Advisor to the Global Neuropsychiatry Group. He conducts expert witness work. None of the other authors has any conflicts of interest to disclose. We confirm that we have read the Journal's position on issues involved in ethical publication and affirm that this report is consistent with those guidelines.

## Supporting information


Data S1.

## Data Availability

No primary data were collected for this study. The original data extraction is available from the corresponding author on reasonable request.
