# Supplementary material for: Effectiveness of antipsychotic drug therapy for treating psychosis in people with epilepsy: A systematic review
Source: Epilepsia. 2024 Oct 21;65(12):3425–40. doi: 10.1111/epi.18123 (PMC11647423; doi:10.1111/epi.18123)
Supplement: Supplementary file 1 — Data S1. [file EPI-65-3425-s001.docx]

Supplementary Material

## **Supplementary Table 1: PRISMA checklist**


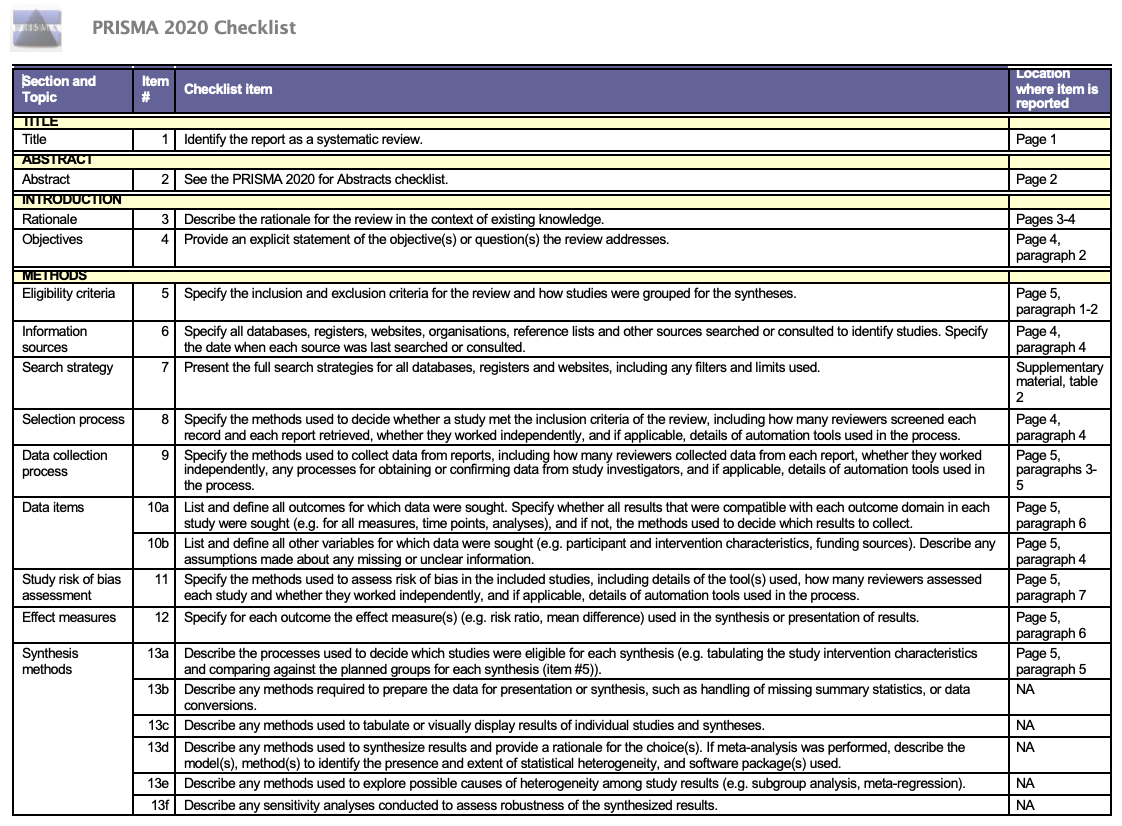


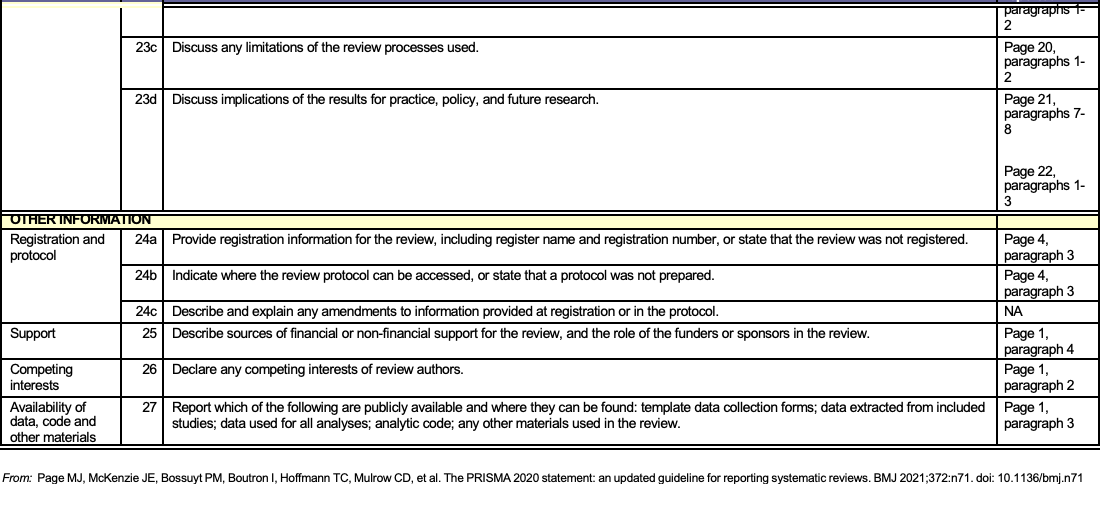

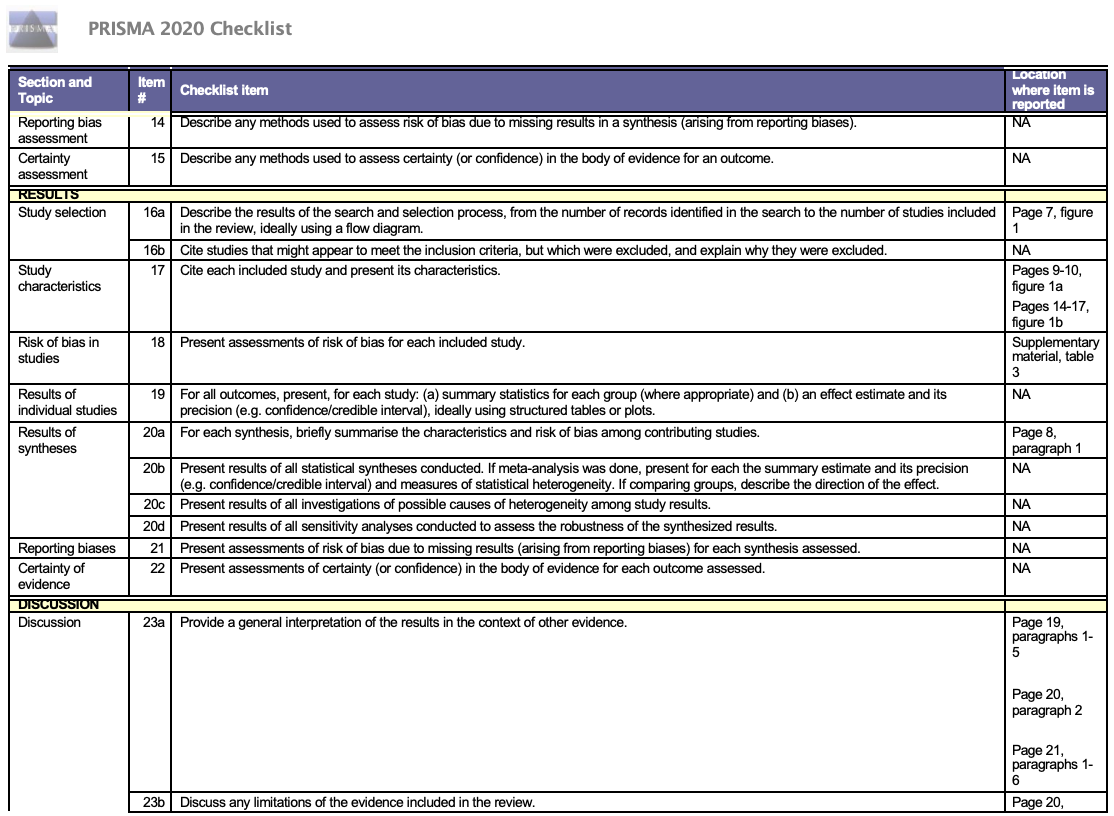


## **Supplementary Table 2: Search strategy**

Database searches were run on 20/06/2023 via Ovid, using a Romper search across MEDLINE, Embase, PsycInfo and AMED.

| Line | Search statement |
| --- | --- |
| 1 | (psychos* or psychotic or hallucination* or delusion* or thought disorder* or schizo*).mp. [mp=title, abstract, heading word, drug trade name, original title, device manufacturer, drug manufacturer, device trade name, keyword heading word, floating subheading word, candidate term word] |
| 2 | exp psychosis/ or exp schizoaffective psychosis/ or exp depressive psychosis/ or exp paranoid psychosis/ or exp manic psychosis/ or exp drug induced psychosis/ or exp endogenous psychosis/ or exp acute psychosis/ or exp affective psychosis/ or exp childhood psychosis/ or exp catatonic schizophrenia/ or exp schizophrenia spectrum disorder/ or exp residual schizophrenia/ or exp latent schizophrenia/ or exp schizophrenia/ or exp schizophrenia assessment/ or exp treatment-resistant schizophrenia/ or exp simple schizophrenia/ or exp paranoid schizophrenia/ |
| 3 | exp persecutory delusion/ or exp somatic delusion/ or exp delusion/ or exp jealous delusion/ or exp grandiose delusion/ or exp delusional disorder/ or exp Capgras syndrome/ or exp delusional misidentification/ |
| 4 | exp thought disorder/ |
| 5 | exp hallucination/ or exp gustatory hallucination/ or exp auditory hallucination/ or exp visual hallucination/ or exp olfactory hallucination/ |
| 6 | (antipsychotic* or neuroleptic* or tranquilis* or phenothiazines or chlorpromazine or levomepromazine or promazine or acepromazine or triflupromazine or cyamemazine or chlorproethazine or piperazine or Dixyrazine or Fluphenazine or Perphenazine or Prochlorperazine or Thiopropazate or Trifluoperazine or Acetophenazine or Thioproperazine or Butaperazine or Perazine or butyrophenone or Periciazine or Thioridazine or Mesoridazine or Pipotiazine or indole or Haloperidol or Trifluperidol or Melperone or Moperone or Pipamperone or Bromperidol or Benperidol or Droperidol or Fluanisone or Lumateperone or Isoindole or thioxanthene or Oxypertine or Molindone or Sertindole or Ziprasidone or Lurasidone or diphenylbutylpiperidine or Flupentixol or Clopenthixol Chlorprothixene or Tiotixene or Zuclopenthixol or Oxepine or benzamide or Fluspirilene or Pimozide or Penfluridol or Loxapine or Clozapine or Olanzapine or Quetiapine or Asenapine or Clotiapine or Olanzapine or Sulpiride or Sultopride or Tiapride or Remoxipride or Amisulpride or Veralipride or Levosulpiride or Lithium or Prothipendyl or Risperidone or Mosapramine or Zotepine or Aripiprazole or Paliperidone or Iloperidone or Cariprazine or Brexpiprazole or Pimavanserin.mp. |
| 7 | exp neuroleptic agent/ or exp atypical antipsychotic agent/ or exp phenothiazines/ or exp chlorpromazine/ or exp levomepromazine/ or exp promazine/ or exp acepromazine/ or exp triflupromazine/ or exp cyamemazine/ or exp chlorproethazine/ or exp piperazine/ or exp Dixyrazine/ or exp Fluphenazine/ or exp Perphenazine/ or exp Prochlorperazine/ or exp Thiopropazate/ or exp Trifluoperazine/ or exp Acetophenazine/ or exp Thioproperazine/ or exp Butaperazine/ or exp Perazine/ or exp butyrophenone/ or exp Periciazine/ or exp Thioridazine/ or exp Mesoridazine/ or exp Pipotiazine/ or exp indole/ or exp Haloperidol/ or exp Trifluperidol/ or exp Melperone/ or exp Moperone/ or exp Pipamperone/ or exp Bromperidol/ or exp Benperidol/ or exp Droperidol/ or exp Fluanisone/ or exp Lumateperone/ or exp Isoindole/ or exp thioxanthene/ or exp Oxypertine/ or exp Molindone/ or exp Sertindole/ or exp Ziprasidone/ or exp Lurasidone/ or exp diphenylbutylpiperidine/ or exp Flupentixol/ or exp Clopenthixol/ or exp Chlorprothixene/ or exp Tiotixene/ or exp Zuclopenthixol/ or exp benzamide/ or exp Fluspirilene/ or exp Pimozide/ or exp Penfluridol/ or exp Loxapine/ or exp Clozapine/ or exp Olanzapine/ or exp Quetiapine/ or exp Asenapine/ or exp Clotiapine/ or exp Olanzapine/ or exp Sulpiride/ or exp Sultopride/ or exp Tiapride/ or exp Remoxipride/ or exp Amisulpride/ or exp Veralipride/ or exp Levosulpiride/ or exp Lithium/ or exp Prothipendyl/ or exp Risperidone/ or exp Mosapramine/ or exp Zotepine/ or exp Aripiprazole/ or exp Paliperidone/ or exp Iloperidone/ or exp Cariprazine/ or exp Brexpiprazole/ or exp Pimavanserin/ or exp oxepine derivative/ |
| 8 | (epilep* or post?ictal or ictal or SLPE or forced normali$ation or alternative or inter?ictal).mp. [mp=title, abstract, heading word, drug trade name, original title, device manufacturer, drug manufacturer, device trade name, keyword heading word, floating subheading word, candidate term word] |
| 9 | exp myoclonus epilepsy/ or exp myoclonic astatic epilepsy/ or exp generalized epilepsy/ or exp startle epilepsy/ or exp drug resistant epilepsy/ or exp traumatic epilepsy/ or exp photosensitive epilepsy/ or exp childhood absence epilepsy/ or exp catamenial epilepsy/ or exp benign childhood epilepsy/ or exp rolandic epilepsy/ or exp temporal lobe epilepsy/ or exp "seizure, epilepsy and convulsion"/ or exp frontal lobe epilepsy/ or exp epilepsy/ or exp lateral temporal lobe epilepsy/ or exp reflex epilepsy/ or exp grand mal epilepsy/ or exp intractable epilepsy/ or exp symptomatic epilepsy/ or exp mesial temporal lobe epilepsy/ or exp focal epilepsy/ |
| 10 | Alternative psychosis.mp |
| 11 | 1 or 2 or 3 or 4 or 5 |
| 12 | 6 or 7 |
| 13 | 8 or 9 |
| 14 | (11 and 13) or 10 |
| 15 | 14 and 12 |
| 16 | 15 use emczd |
|  |  |
| 17 | exp Schizophrenia/ or exp Psychoses, Substance-Induced/ or (psychos* or schizo*).mp. |
| 18 | exp Psychotic Disorders/ or psychot*.mp. |
| 19 | exp somatic delusion/ or exp Delusions/ or exp jealous delusion/ or exp grandiose delusion/ or exp delusional disorder/ or exp Capgras syndrome/ or exp delusional misidentification/ or (delusion* or persecutory delusion*).mp. |
| 20 | exp psychosis/ or exp paranoid psychosis/ or exp manic psychosis/ or exp drug induced psychosis/ or exp endogenous psychosis/ or exp acute psychosis/ or exp affective psychosis/ or exp childhood psychosis/ or exp catatonic schizophrenia/ or exp schizophrenia spectrum disorder/ or exp residual schizophrenia/ or exp latent schizophrenia/ or exp schizophrenia/ or exp schizophrenia assessment/ or exp treatment-resistant schizophrenia/ or exp simple schizophrenia/ or exp paranoid schizophrenia/ or exp chronic psychosis/ or exp functional psychosis/ or exp organic psychosis/ or exp secondary psychosis/ or exp primary psychosis/ |
| 21 | thought disorder*.mp. |
| 22 | exp hallucination/ or exp gustatory hallucination/ or exp auditory hallucination/ or exp visual hallucination/ or exp olfactory hallucination/ or exp hypnagogic hallucination/ or hallucinat*.mp. |
| 23 | 17 or 18 or 19 or 20 or 21 or 22 |
| 24 | (antipsychotic* or neuroleptic* or tranquilis* or phenothiazines or chlorpromazine or levomepromazine or promazine or acepromazine or triflupromazine or cyamemazine or chlorproethazine or piperazine or Dixyrazine or Fluphenazine or Perphenazine or Prochlorperazine or Thiopropazate or Trifluoperazine or Acetophenazine or Thioproperazine or Butaperazine or Perazine or butyrophenone or Periciazine or Thioridazine or Mesoridazine or Pipotiazine or indole or Haloperidol or Trifluperidol or Melperone or Moperone or Pipamperone or Bromperidol or Benperidol or Droperidol or Fluanisone or Lumateperone or Isoindole or thioxanthene or Oxypertine or Molindone or Sertindole or Ziprasidone or Lurasidone or diphenylbutylpiperidine or Flupentixol or Clopenthixol Chlorprothixene or Tiotixene or Zuclopenthixol or Oxepine or benzamide or Fluspirilene or Pimozide or Penfluridol or Loxapine or Clozapine or Olanzapine or Quetiapine or Asenapine or Clotiapine or Olanzapine or Sulpiride or Sultopride or Tiapride or Remoxipride or Amisulpride or Veralipride or Levosulpiride or Lithium or Prothipendyl or Risperidone or Mosapramine or Zotepine or Aripiprazole or Paliperidone or Iloperidone or Cariprazine or Brexpiprazole or Pimavanserin).mp. |
| 25 | exp neuroleptic agent/ or exp phenothiazines/ or exp chlorpromazine/ or exp levomepromazine/ or exp promazine/ or exp acepromazine/ or exp triflupromazine/ or exp chlorproethazine/ or exp piperazine/ or exp Dixyrazine/ or exp Fluphenazine/ or exp Perphenazine/ or exp Prochlorperazine/ or exp Thiopropazate/ or exp Trifluoperazine/ or exp Acetophenazine/ or exp Thioproperazine/ or exp Butaperazine/ or exp Perazine/ or exp butyrophenone/ or exp Periciazine/ or exp Thioridazine/ or exp Mesoridazine/ or exp Pipotiazine/ or exp indole/ or exp Haloperidol/ or exp Trifluperidol/ or exp Melperone/ or exp Moperone/ or exp Pipamperone/ or exp Bromperidol/ or exp Benperidol/ or exp Droperidol/ or exp Fluanisone/ or exp Lumateperone/ or exp Isoindole/ or exp thioxanthene/ or exp Oxypertine/ or exp Molindone/ or exp Sertindole/ or exp Ziprasidone/ or exp Lurasidone/ or exp diphenylbutylpiperidine/ or exp Flupentixol/ or exp Clopenthixol/ or exp Chlorprothixene/ or exp Tiotixene/ or exp Zuclopenthixol/ or exp benzamide/ or exp Fluspirilene/ or exp Pimozide/ or exp Penfluridol/ or exp Loxapine/ or exp Clozapine/ or exp Olanzapine/ or exp Quetiapine/ or exp Asenapine/ or exp Clotiapine/ or exp Olanzapine/ or exp Sulpiride/ or exp Sultopride/ or exp Tiapride/ or exp Remoxipride/ or exp Amisulpride/ or exp Veralipride/ or exp Levosulpiride/ or exp Lithium/ or exp Prothipendyl/ or exp Risperidone/ or exp Mosapramine/ or exp Zotepine/ or exp Aripiprazole/ or exp Paliperidone/ or exp Iloperidone/ or exp Cariprazine/ or exp Brexpiprazole/ or exp Pimavanserin/ or exp oxepine derivative/ |
| 26 | 24 or 25 |
| 27 | exp myoclonus epilepsy/ or exp myoclonic astatic epilepsy/ or exp generalized epilepsy/ or exp drug resistant epilepsy/ or exp traumatic epilepsy/ or exp photosensitive epilepsy/ or exp childhood absence epilepsy/ or exp benign childhood epilepsy/ or exp rolandic epilepsy/ or exp temporal lobe epilepsy/ or exp "seizure, epilepsy and convulsion"/ or exp frontal lobe epilepsy/ or exp epilepsy/ or exp lateral temporal lobe epilepsy/ or exp reflex epilepsy/ or exp grand mal epilepsy/ or exp intractable epilepsy/ or exp symptomatic epilepsy/ or exp mesial temporal lobe epilepsy/ or exp focal epilepsy/ or exp epilepsies, partial/ or exp epilepsy, temporal lobe/ or exp seizures/ or catamenial epilepsy.mp. |
| 28 | epilep*.mp. |
| 29 | post?ictal.mp. |
| 30 | ictal.mp. |
| 31 | SLPE.mp. or exp Schizophrenic Psychology/ |
| 32 | inter?ictal.mp. |
| 33 | Alternative psychosis.mp |
| 34 | 27 or 28 or 29 or 30 or 31 or 32 |
| 35 | (23 AND 34) OR 33 |
| 36 | 26 AND 35 |
| 37 | 36 use medall |
|  |  |
| 38 | (psychos* or psychotic or hallucination* or delusion* or thought disorder* or schizo*).mp. |
| 39 | exp Reactive Psychosis/ or exp Childhood Onset Psychosis/ or exp Affective Psychosis/ or exp Paranoid Psychosis/ or exp Chronic Psychosis/ or exp Psychosis/ |
| 40 | exp delusions/ |
| 41 | exp thought disorders/ |
| 42 | exp Drug Induced Hallucinations/ or exp Visual Hallucinations/ or exp Auditory Hallucinations/ or exp Hypnagogic Hallucinations/ or exp Hallucinations/ |
| 43 | (antipsychotic* or neuroleptic*or tranquilis* or phenothiazines or chlorpromazine or levomepromazine or promazine or acepromazine or triflupromazine or cyamemazine or chlorproethazine or piperazine or Dixyrazine or Fluphenazine or Perphenazine or Prochlorperazine or Thiopropazate or Trifluoperazine or Acetophenazine or Thioproperazine or Butaperazine or Perazine or butyrophenone or Periciazine or Thioridazine or Mesoridazine or Pipotiazine or indole or Haloperidol or Trifluperidol or Melperone or Moperone or Pipamperone or Bromperidol or Benperidol or Droperidol or Fluanisone or Lumateperone or Isoindole or thioxanthene or Oxypertine or Molindone or Sertindole or Ziprasidone or Lurasidone or diphenylbutylpiperidine or Flupentixol or Clopenthixol Chlorprothixene or Tiotixene or Zuclopenthixol or Oxepine or benzamide or Fluspirilene or Pimozide or Penfluridol or Loxapine or Clozapine or Olanzapine or Quetiapine or Asenapine or Clotiapine or Olanzapine or Sulpiride or Sultopride or Tiapride or Remoxipride or Amisulpride or Veralipride or Levosulpiride or Lithium or Prothipendyl or Risperidone or Mosapramine or Zotepine or Aripiprazole or Paliperidone or Iloperidone or Cariprazine or Brexpiprazole or Pimavanserin).mp. |
| 44 | exp Neuroleptic Drugs/ or exp phenothiazine derivatives/ or exp chlorpromazine/ or exp levomepromazine/ or exp promazine/ or exp acepromazine/ or exp triflupromazine/ or exp cyamemazine/ or exp chlorproethazine/ or exp piperazine/ or exp Dixyrazine/ or exp Fluphenazine/ or exp Perphenazine/ or exp Prochlorperazine/ or exp Thiopropazate/ or exp Trifluoperazine/ or exp Acetophenazine/ or exp Thioproperazine/ or exp Butaperazine/ or exp Perazine/ or exp butyrophenone/ or exp Periciazine/ or exp Thioridazine/ or exp Mesoridazine/ or exp Pipotiazine/ or exp indole/ or exp Haloperidol/ or exp Trifluperidol/ or exp Melperone/ or exp Moperone/ or exp Pipamperone/ or exp Bromperidol/ or exp Benperidol/ or exp Droperidol/ or exp Fluanisone/ or exp Lumateperone/ or exp Isoindole/ or exp thioxanthene/ or exp Oxypertine/ or exp Molindone/ or exp Sertindole/ or exp Ziprasidone/ or exp Lurasidone/ or exp diphenylbutylpiperidine/ or exp Flupentixol/ or exp Clopenthixol/ or exp Chlorprothixene/ or exp Tiotixene/ or exp Zuclopenthixol/ or exp benzamide/ or exp Fluspirilene/ or exp Pimozide/ or exp Penfluridol/ or exp Loxapine/ or exp Clozapine/ or exp Olanzapine/ or exp Quetiapine/ or exp Asenapine/ or exp Clotiapine/ or exp Olanzapine/ or exp Sulpiride/ or exp Sultopride/ or exp Tiapride/ or exp Remoxipride/ or exp Amisulpride/ or exp Veralipride/ or exp Levosulpiride/ or exp Lithium/ or exp Prothipendyl/ or exp Risperidone/ or exp Mosapramine/ or exp Zotepine/ or exp Aripiprazole/ or exp Paliperidone/ or exp Iloperidone/ or exp Cariprazine/ or exp Brexpiprazole/ or exp Pimavanserin/ or exp oxepine derivative/ |
| 45 | (epilep* or post?ictal or ictal or SLPE or forced normali$ation or inter?ictal).mp. [mp=title, abstract, heading word, drug trade name, original title, device manufacturer, drug manufacturer, device trade name, keyword heading word, floating subheading word, candidate term word] |
| 46 | exp Seizures/ or exp Anticonvulsive Drugs/ or exp Epilepsy/ or exp Epileptic Seizures/ |
| 47 | Alternative psychosis.mp |
| 48 | 38 or 39 or 40 or 41 or 42 |
| 49 | 43 OR 44 |
| 50 | 45 or 46 |
| 51 | (48 AND 50) OR 47 |
| 52 | 51 AND 49 |
| 53 | 52 use psycinfo |
|  |  |
| 54 | psychos*.mp. [mp=abstract, heading words, title] |
| 55 | psychot*.mp. [mp=abstract, heading words, title] |
| 56 | exp schizophrenia/ or exp psychotic disorders/ or exp psychosis/ or exp depressive psychosis/ or exp paranoid psychosis/ or exp manic psychosis/ or exp drug induced psychosis/ or exp endogenous psychosis/ or exp acute psychosis/ or exp affective psychosis/ or exp childhood psychosis/ or exp catatonic schizophrenia/ or exp schizophrenia spectrum disorder/ or exp residual schizophrenia/ or exp latent schizophrenia/ or exp schizophrenia/ or exp schizophrenia assessment/ or exp treatment-resistant schizophrenia/ or exp simple schizophrenia/ or exp paranoid schizophrenia/ or exp chronic psychosis/ or schizo*.mp. |
| 57 | (schizophrenia or substance-induced psychoses or psychotic disorders or psychosis or depressive psychosis or paranoid psychosis or manic psychosis or drug induced psychosis or endogenous psychosis or acute psychosis or affective psychosis or childhood psychosis or catatonic schizophrenia or schizophrenia spectrum disorder or residual schizophrenia or latent schizophrenia or schizophrenia or schizophrenia assessment or treatment-resistant schizophrenia or simple schizophrenia or paranoid schizophrenia or chronic psychosis).mp. |
| 58 | thought disorder*.mp. |
| 59 | exp persecutory delusion/ or exp somatic delusion/ or exp Delusions/ or exp jealous delusion/ or exp grandiose delusion/ or exp delusional disorder/ or exp Capgras syndrome/ or exp delusional misidentification/ or delusion*.mp. |
| 60 | exp exp Hallucinations/ or exp gustatory hallucination/ or exp auditory hallucination/ or exp visual hallucination/ or exp olfactory hallucination/ or hallucinat*.mp. |
| 61 | 54 or 55 or 56 or 57 or 58 or 59 or 60 |
| 62 | (antipsychotic* or neuroleptic* or tranquilis* or phenothiazines or chlorpromazine or levomepromazine or promazine or acepromazine or triflupromazine or cyamemazine or chlorproethazine or piperazine or Dixyrazine or Fluphenazine or Perphenazine or Prochlorperazine or Thiopropazate or Trifluoperazine or Acetophenazine or Thioproperazine or Butaperazine or Perazine or butyrophenone or Periciazine or Thioridazine or Mesoridazine or Pipotiazine or indole or Haloperidol or Trifluperidol or Melperone or Moperone or Pipamperone or Bromperidol or Benperidol or Droperidol or Fluanisone or Lumateperone or Isoindole or thioxanthene or Oxypertine or Molindone or Sertindole or Ziprasidone or Lurasidone or diphenylbutylpiperidine or Flupentixol or Clopenthixol Chlorprothixene or Tiotixene or Zuclopenthixol or Oxepine or benzamide or Fluspirilene or Pimozide or Penfluridol or Loxapine or Clozapine or Olanzapine or Quetiapine or Asenapine or Clotiapine or Olanzapine or Sulpiride or Sultopride or Tiapride or Remoxipride or Amisulpride or Veralipride or Levosulpiride or Lithium or Prothipendyl or Risperidone or Mosapramine or Zotepine or Aripiprazole or Paliperidone or Iloperidone or Cariprazine or Brexpiprazole or Pimavanserin).mp. |
| 63 | exp atypical antipsychotic/ or exp tranquilising agent/ or exp phenothiazines/ or exp chlorpromazine/ or exp levomepromazine/ or exp promazine/ or exp acepromazine/ or exp triflupromazine/ or exp cyamemazine/ or exp chlorproethazine/ or exp piperazine/ or exp Dixyrazine/ or exp Fluphenazine/ or exp Perphenazine/ or exp Prochlorperazine/ or exp Thiopropazate/ or exp Trifluoperazine/ or exp Acetophenazine/ or exp Thioproperazine/ or exp Butaperazine/ or exp Perazine/ or exp butyrophenone/ or exp Periciazine/ or exp Thioridazine/ or exp Mesoridazine/ or exp Pipotiazine/ or exp indole/ or exp Haloperidol/ or exp Trifluperidol/ or exp Melperone/ or exp Moperone/ or exp Pipamperone/ or exp Bromperidol/ or exp Benperidol/ or exp Droperidol/ or exp Fluanisone/ or exp Lumateperone/ or exp Isoindole/ or exp thioxanthene/ or exp Oxypertine/ or exp Molindone/ or exp Sertindole/ or exp Ziprasidone/ or exp Lurasidone/ or exp diphenylbutylpiperidine/ or exp Flupentixol/ or exp Clopenthixol/ or exp Chlorprothixene/ or exp Tiotixene/ or exp Zuclopenthixol/ or exp benzamide/ or exp Fluspirilene/ or exp Pimozide/ or exp Penfluridol/ or exp Loxapine/ or exp Clozapine/ or exp Olanzapine/ or exp Quetiapine/ or exp Asenapine/ or exp Clotiapine/ or exp Olanzapine/ or exp Sulpiride/ or exp Sultopride/ or exp Tiapride/ or exp Remoxipride/ or exp Amisulpride/ or exp Veralipride/ or exp Levosulpiride/ or exp Lithium/ or exp Prothipendyl/ or exp Risperidone/ or exp Mosapramine/ or exp Zotepine/ or exp Aripiprazole/ or exp Paliperidone/ or exp Iloperidone/ or exp Cariprazine/ or exp Brexpiprazole/ or exp Pimavanserin/ or exp oxepine derivative/ |
| 64 | 62 or 63 |
| 65 | exp myoclonus epilepsy/ or exp myoclonic astatic epilepsy/ or exp generalized epilepsy/ or exp startle epilepsy/ or exp drug resistant epilepsy/ or exp traumatic epilepsy/ or exp photosensitive epilepsy/ or exp childhood absence epilepsy/ or exp catamenial epilepsy/ or exp benign childhood epilepsy/ or exp rolandic epilepsy/ or exp temporal lobe epilepsy/ or exp "seizure, epilepsy and convulsion"/ or exp frontal lobe epilepsy/ or exp epilepsy/ or exp lateral temporal lobe epilepsy/ or exp reflex epilepsy/ or exp grand mal epilepsy/ or exp intractable epilepsy/ or exp symptomatic epilepsy/ or exp mesial temporal lobe epilepsy/ or exp focal epilepsy/ or exp epilepsies, partial/ or exp epilepsy, temporal lobe/ or exp seizures/ |
| 66 | epilep*.mp. [mp=abstract, heading words, title] |
| 67 | post?ictal.mp. [mp=abstract, heading words, title] |
| 68 | ictal.mp. [mp=abstract, heading words, title] |
| 69 | SLPE.mp. |
| 70 | inter?ictal.mp. [mp=abstract, heading words, title] |
| 71 | Alternative psychosis.mp |
| 72 | 65 or 66 or 67 or 68 or 69 or 70 |
| 73 | (61 AND 72) OR 71 |
| 74 | 73 AND 64 |
| 75 | 74 use amed |
| 76 | 16 or 37 or 53 or 75 |

The OpenGrey database was searched using the following terms “Epilep* AND Psycho* and Antipsychotic*”

## **Supplementary Table 3a: Study Characteristics- Case Series**

**Case series**

| **Study** | **Location** | **Method of recruitment** | **Inclusion Criteria** | **Exclusion Criteria** |
| --- | --- | --- | --- | --- |
| [Blumer et al., 2000](https://www.zotero.org/google-docs/?broken=SkGoQW)^37^ | United States | Hospitalised patients | Not explicitly stated  Only patients experiencing psychosis and epilepsy were included | Not stated |
| [Langosch](https://www.zotero.org/google-docs/?broken=faP5mC) et al., 2002^38^ | United Kingdom | Hospitalised patients | Not explicitly stated  Only patients that had severe treatment resistant psychosis were recruited | Not stated |
| [Leinonen](https://www.zotero.org/google-docs/?broken=1dID1v) et al., 2009^18^ | Finland | Hospitalised patients | Adult patients with intractable epilepsy who underwent temporal lobe surgery and developed postoperative psychotic illness during the 1 year postoperative follow-up | Not stated |
| [Pakalnis et al., 1987](https://www.zotero.org/google-docs/?broken=tO1ppS)^19^ | United States | Hospitalised Patients | Epileptic patients who developed acute psychotic states on establishment of seizure control and normalization of previously abnormal electroencephalograms with frank epileptiform activity, normal results of neurologic and mental status examinations and normal computed tomographic (CT) brain scans. | Previous psychiatric history |

## **Supplementary Table 3b: Study Characteristics- Cohort Studies**

**Cohort studies**

| **Study** | **Location** | **Method of recruitment** | **Inclusion Criteria** | **Exclusion Criteria** |
| --- | --- | --- | --- | --- |
| Adachi et al., 2012^43^ | Japan | Hospitalised patients | Interictal psychosis diagnosis  Capacity assessment for treatment  Clinical need as decided by the clinician | Progressive brain disease, dementing process or comorbid substance misuse |
|  |  |  |  |  |
|  |  |  |  |  |
| Adachi et al., 2007^39^ | Japan | Hospitalised patients | Postictal psychosis diagnosis between Jan 1980 and Dec 2005. | Episodes of psychosis related to nonconvulsive status epilepticus or antiepileptic drug toxicity Included: - patients  PIP episodes not adequately observed or for  which records were incomplete |
|  |  |  |  |  |
|  |  |  |  |  |
| Chen et al., 2016^17^ | Australia | Hospitalised patients | Onset of the psychotic disorder at 16 years or older  Diagnosis of epilepsy  Admission to hospital or attendance at epilepsy outpatient clinic for psychotic symptoms | Psychogenic non-epileptic seizures   Psychotic symptoms as part of the ictal semiology  An organic illness with known psychiatric manifestations, e.g. Wilson’s disease. |
|  |  |  |  |  |
|  |  |  |  |  |
|  |  |  |  |  |
| Hamed et al., 2019^44^ | Egypt | Hospitalised patients | Adults with epilepsy and psychosis | Age at examination less than 20 years old  Remote symptomatic or secondary epilepsy (other than HS in patients with TLE) or an epilepsy syndrome  Other medical or neurologic diseases  Premorbid psychiatric illness or personality disorder  History of brain surgery, substance abuse  Borderline or low intelligence (<70 as assessed by Wechsler Intelligence Scale for adults 3rd edition or WISA-III)   Lack of compliance to AEDs therapy in the last 6 months before participation in the study |
|  |  |  |  |  |
| Hara et al., 2013^40^ | Japan | Hospitalised patients | Patients with inter-ictal psychosis | Patients with progressive brain diseases, dementing processes, or substance misuse |
|  |  |  |  |  |
|  |  |  |  |  |
|  |  |  |  |  |
|  |  |  |  |  |
| Kara et al., 2017^45^ | Turkey | Hospitalised patients | Epileptic patients with postictal psychiatric symptoms | Children or pregnant women  Patients with an intracranial mass, substance abuse  Poststroke epilepsy  Status epilepticus  Previous shunt operation  Seizures secondary to infection or sepsis, electrolyte imbalance, hydrocephalus, alcohol abuse, and pseudo-epilepsy, or drug overdoses |
|  |  |  |  |  |
|  |  |  |  |  |
| [Kubagawa](https://www.zotero.org/google-docs/?broken=z8y3Vk) et al., 1997^46^ | Japan | Hospitalised patients | 23 formerly epileptic patients who developed psychosis after their AEDs were successfully dis- continued | 21.94, 8.86  3M, 13F |
|  |  |  |  |  |
| Okazaki et al., 2014^41^ | Japan | Hospitalised patients | Epilepsy and psychiatric symptoms, including psychosis | Progressive neurodegenerative disease or dementing process  Developing brain mass lesion requiring surgical treatments  Substance misuse |
|  |  |  |  |  |
| Tadokoro et al., 2007^42^ | Japan | Hospitalised patients | Diagnosis of epilepsy and either inter-ictal psychosis or schizophrenia | Patients with previous or ongoing psychotic episodes were excluded from the beginning of the present study |
|  |  |  |  |  |

## **Supplementary Table 4a: Risk of Bias Assessment- Case Series**

Case series

| Study | Were there clear criteria for inclusion in the case series? | Was the condition measured in a standard, reliable way for all participants included in the case series? | Were valid methods used for identification of the condition for all participants included in the case series? | Did the case series have consecutive inclusion of participants? | Did the case series have complete inclusion of participants? | Was there clear reporting of the demographics of the participants in the study? | Was there clear reporting of clinical information of the participants? | Were the outcomes or follow up results of cases clearly reported? | Was there clear reporting of the presenting site(s)/clinic(s) demographic information? | Was statistical analysis appropriate? | Score | Overall appraisal |
| --- | --- | --- | --- | --- | --- | --- | --- | --- | --- | --- | --- | --- |
| [Blumer et al., 2000](https://www.zotero.org/google-docs/?broken=SkGoQW)18 | Y | N | Y | N | N | Y | N | Y | Y | Y | 6/10 | Moderate |
| [Langosch](https://www.zotero.org/google-docs/?broken=faP5mC) et al., 200220 | Y | U | U | U | Y | Y | Y | U | N | Y | 5/10 | Moderate |
| [Leinonen](https://www.zotero.org/google-docs/?broken=1dID1v) et al., 200919 | Y | Y | Y | Y | Y | Y | Y | Y | N | Y | 9/10 | Low |
| [Pakalnis et al., 1987](https://www.zotero.org/google-docs/?broken=tO1ppS)21 | U | N | U | U | U | Y | Y | U | N | NA | 2/9 | High |

Case Series: 10 criteria. Y = yes, N = no, U = unclear, NA = not applicable. Risk of bias calculation: Y = 1, N = 0. Overall risk of bias appraisal: High = score≤33%, Moderate = score≤66%, Low = score>66%

## **Supplementary Table 4b: Risk of Bias Assessment- Cohort Studies**

Cohort studies

| Study | Were the two groups similar and recruited from the same population? | Were the exposures measured similarly to assign people to both exposed and unexposed groups? | Was the exposure measured in a valid and reliable way? | Were confounding factors identified? | Were strategies to deal with confounding factors stated? | Were the groups/participants free of the outcome at the start of the study (or at the moment of exposure)? | Were the outcomes measured in a valid and reliable way? | Was the follow up time reported and sufficient to be long enough for outcomes to occur? | Was follow up complete, and if not, were the reasons to loss to follow up described and explored? | Were strategies to address incomplete follow up utilized? | Was appropriate statistical analysis used? | Score | Overall appraisal |
| --- | --- | --- | --- | --- | --- | --- | --- | --- | --- | --- | --- | --- | --- |
| Adachi et al., 201226 | Y | U | Y | U | U | Y | Y | Y | Y | U | Y | 7/11 | Moderate |
| Adachi et al., 200728 | Y | Y | Y | U | N | Y | U | Y | Y | Y | Y | 8/11 | Low |
| Chen et al., 201629 | Y | Y | Y | Y | N | Y | Y | Y | Y | NA | Y | 9/10 | Low |
| Hamed et al., 201922 | Y | Y | Y | N | NA | N | Y | U | Y | U | Y | 6/10 | Moderate |
| Hara et al., 201327 | Y | Y | Y | N | N | Y | U | Y | Y | NA | Y | 7/10 | Low |
| Kara et al., 201730 | Y | Y | Y | Y | N | Y | Y | U | Y | NA | Y | 6/10 | Moderate |
| [Kubagawa](https://www.zotero.org/google-docs/?broken=z8y3Vk) et al., 199723 | Y | Y | Y | N | N | Y | U | Y | Y | NA | U | 6/10 | Moderate |
| Okazaki et al., 201424 | Y | Y | Y | Y | N | Y | Y | Y | Y | U | Y | 9/11 | Low |
| Tadokoro et al., 200725 | N | Y | Y | Y | N | Y | Y | Y | N | Y | Y | 8/11 | Low |

# Cohort studies: 11 criteria. Y = yes, N = no, U = unclear, NA = not applicable. Risk of bias calculation: Y = 1, N = 0. Overall risk of bias appraisal: High = score≤33%, Moderate = score≤66%, Low = score>66%
